# Investigation of the Anti-Prostate Cancer Properties of Marine-Derived Compounds

**DOI:** 10.3390/md16050160

**Published:** 2018-05-12

**Authors:** Meiqi Fan, Amit Kumar Nath, Yujiao Tang, Young-Jin Choi, Trishna Debnath, Eun-Ju Choi, Eun-Kyung Kim

**Affiliations:** 1 Division of Food Bioscience, College of Biomedical and Health Sciences, Konkuk University, Chungju 27478, Korea; fanmeiqi@kku.ac.kr (M.F.); chinmoyamit@gmail.com (A.K.N.); yuanxi00@126.com (Y.T.); choijang11@kku.ac.kr (Y.-J.C.); 2 School of Bio-Science and Food Engineering, Changchun University of Science and Technology, Changchun 130-600, China; 3 Department of Food Science and Biotechnology, Dongguk University, Goyang 10326, Korea; trishna_rahul@yahoo.com; 4 Department of Physical Education, College of Education, Daegu Catholic University, Gyeongsan 38430, Korea; cej0915@cu.ac.kr

**Keywords:** prostate cancer, antioxidant, anti-proliferative, apoptosis, natural marine compounds

## Abstract

This review focuses on marine compounds with anti-prostate cancer properties. Marine species are unique and have great potential for the discovery of anticancer drugs. Marine sources are taxonomically diverse and include bacteria, cyanobacteria, fungi, algae, and mangroves. Marine-derived compounds, including nucleotides, amides, quinones, polyethers, and peptides are biologically active compounds isolated from marine organisms such as sponges, ascidians, gorgonians, soft corals, and bryozoans, including those mentioned above. Several compound classes such as macrolides and alkaloids include drugs with anti-cancer mechanisms, such as antioxidants, anti-angiogenics, antiproliferatives, and apoptosis-inducing drugs. Despite the diversity of marine species, most marine-derived bioactive compounds have not yet been evaluated. Our objective is to explore marine compounds to identify new treatment strategies for prostate cancer. This review discusses chemically and pharmacologically diverse marine natural compounds and their sources in the context of prostate cancer drug treatment.

## 1. Introduction

Throughout the history of humanity, marine sources have played an important role as a source of natural medicinal products. The ocean covers almost 70% of the earth’s surface and contains varied environmental conditions. Half of the previously described novel marine natural compounds have been shown to be biologically active [1]. The marine ecological system is unique. Therefore, marine organisms must survive and adapt to these harsh environmental conditions. Researchers have become increasingly interested in marine sources in the development of potential anticancer drugs [2].

Cancer is the most devastating disease in recent years. According to a report on prostate cancer from Research Fund International, 1.1 million prostate cancer cases were reported in 2012, corresponding to 8% of all new cancer patients and 15% of male cancer patients. Prostate cancer is one of the most common malignant diseases in men but is primarily seen in developed countries. Prostate cancer is the second-most common cause of death among all male cancer patients [3].

Prostate cancer can spread to other parts of the body, especially the bones and lymph nodes [4]. Prostate cancer is an androgen-dependent carcinoma that is mediated through androgen receptor (AR)-regulated genes [5]. The testes produce the most androgens, with a small amount of androgen produced through the conversion of adrenal steroids [6]. In the early stages of prostate cancer, cancerous cells can metastasize through the lymphatic fluid to other organs. Prostate cancer progresses through the activation of growth factors that promote critical signaling of cascades [7].

Marine compounds have shown potential for the treatment of prostate cancer. In this review, we discuss the various species and compounds and their specific effects on prostate cancer. Among the diverse number of invertebrates, sponges have the most significant medicinal importance. Screening of cyanobacteria, fungi, sponges, algae, and tunicates has yielded a large number of anticancer compounds; these marine-derived chemical compounds include alkaloids, macrolides, terpenoids, among other compounds.

## 2. Molecular Targets for Anti-Prostate Cancer Compounds

Prostate cancer is a disease of the prostate characterized by disordered cell growth and proliferation. Mutations of the oncogenes are responsible for prostate cancer. These oncogenes encode altered proteins that result in increased cell growth and proliferation [8]. The proteins encoded by oncogenes include growth factors; receptor tyrosine kinases such as epidermal growth factor receptor (EGFR) and vascular endothelial growth factor receptor (VEGFR); tyrosine kinases including Src family of protein tyrosine kinase (Src) and Bruton’s tyrosine kinase (BTK); serine/threonine kinase families such as rapidly accelerated fibrosarcoma (RAF), cyclin dependent kinase (CDK), and checkpoint kinase (CHK); guanosine triphosphate hydrolase enzymes (GTPases) such as the rat sarcoma (Ras) protein; and various transcription factors including myelocytomatosis oncogene (Myc). Mutation or overexpression of oncogenes leads to aberrant and excessive cell proliferation [9].

Androgens stimulate the growth of prostate cancer cells. Higher levels of androgens might contribute to prostate cancer risk in some men. P53 is a tumor suppressor gene responsible for the control of cell growth and proliferation as well as diminishing AR-mediated signaling in prostate cancer cell lines [10]. Insulin-like growth factor-1 (IGF-1) is associated with the risk of prostate cancer [11]. E-cadherin is an important biomarker for prostate cancer diagnosis and is involved in cell-cell adhesion, which is linked to invasion and metastasis. E-cadherin binds to β-catenin and forms a protein complex to prevent adhesion and migration.

Matrix metalloproteinases (MMP)-1 and MMP-2 play a key role in the metastasis, invasion, and angiogenesis of prostate cancer. MMP-1 and MMP-2 are novel molecular biomarkers and tissue inhibitors of matrix metalloproteinases (TIMPs) responsible for the regulation of angiogenesis and are potentially invasive and metastatic [12].

In addition, overexpression of caveolin-1 (CAV-1), zinc-dependent mammalian histone deacetylase (HDAC), 3-phosphoinositide-dependent protein kinase-1 (PDPK1), PG receptor EP2 and FP, prostaglandin-degrading enzyme (15-PGDH), and prostaglandin-endoperoxide synthase protein cyclooxygenase-2 (COX-2) also trigger the development of prostate cancer [13].

## 3. Bioactive Products with Potential for Prostate Cancer Treatment

Marine bioactive compounds and their biological activity towards prostate cancer are summarized in Table 1.

### 3.1. Marine Bacteria

Marine microorganisms offer a unique source for potential anticancer drugs. Scientists are interested in marine microorganisms for the development of these drugs. Marine microorganisms have yielded novel anti-inflammatory agents such as pseudopterosins, topsentins, scytonemin, manoalide, topsentins, and scytonemin [44]. These agents show cytotoxic activity against cancer cell lines like PC-3 (prostate cancer cells).

Microbes associated with the mollusk *Elysia rufescens* synthesize kahalalide F (KF), a compound with both in vitro and in vivo antitumor activity in various solid tumor models. In vitro antiproliferative studies have found activity in certain prostate cancer cell lines (PC-3, DU-145), but none against the hormone-sensitive LNCaP line [14]. Therefore, KF exhibits antitumor activity against solid prostate tumors []. In clinical trials, adult patients with advanced or metastatic androgen-refractory prostate cancer have received intravenous administration of KF. One patient had a partial response at a dose of 80 μg per kg per day, showing a prostate-specific antigen decline of at least 50% for ≥4 weeks. Five patients showed stable disease. KF can be safely administered as a one-hour infusion for five consecutive days at a dose of 560 μg per kg per day once every three weeks [15].

### 3.2. Marine Fungi

Marine fungi provide a rich profile of biologically active metabolites. Despite the interests toward studying of biologically active metabolites, such studies remain scarce. The effect of marine fungal metabolite 1386A from the South China Sea on the proliferation of androgen-independent cells has been reported in DU-145 cells. The half-maximal inhibitory concentrations (IC_50_) of 1386A incubated with DU-145 cells for 24, 48, and 72 h were 25.31, 8.62, and 4.79 μmol/L respectively [45]. This activity may be useful in diseases including prostate cancer as a therapeutic or food additive [45].

Demethoxyfumitremorgin C, a secondary metabolite of the marine fungus, Aspergillus fumigatus, had been reported to inhibited the cell viability on PC-3 cells [16].

An investigation into new bioactive metabolites of marine gut fungi revealed aspochalasins isolated from the gut of the marine isopod *Ligia oceanica*. Aspochalasins are a subgroup of cytochalasans consisting of a macrocylic ring, isoindolone moiety, and a 2-methyl-propyl side-chain. Aspochalasins showed various bioactivities such as cytotoxicity [17], anti-HIV [46], and TNF-alpha [47] and melanogenesis inhibitors [48]. Cytotoxicity against the prostate cancer PC-3 cell line was assayed using the MTT method. Apochalasin V showed moderate activity at IC_50_ values of 30.4 μM, respectively [17].

### 3.3. Marine Sponges

Marine sponges are an abundant source of alkaloids. For example, rhizochalin is a bioactive substance initially isolated from the marine sponge *Rhizochalina incrustata*. Rhizochalin exhibited anticancer properties in human castration-resistant prostate cancer cells, induced apoptosis, and G2/M cell cycle arrest, and inhibited autophagy [18]. Rhizochalinin (Rhiz) is a sphingolipid-like semi-synthetic compound hydrolytically derived from rhizochalin. Rhiz had cytotoxic effects on all human prostate cancer cell lines (PC-3, DU145, LNCaP, 22Rv1, VCaP) at low micromolar concentrations. In general, aglycones are more cytotoxic than glycosides [19]. Functional analyses confirmed an anti-migratory effect of Rhiz in PC-3 cells. Additionally, a predicted ERK1/2 activation was confirmed by Western blot analysis, and the prosurvival effects in Rhiz-treated prostate cancer cells indicated a potential mechanism of resistance [49].

In addition, two prominent alkaloids, heliclonadiamines (HCA) from ethanol extracts of the marine sponge *Haliclona* spp. show strong cytotoxic effects against PC-3 cells, with 50% viability at 100 μM [50]. Overexpression of phosphatase of regenerating liver-3 (PRL-3) in these cells was suppressed by treatment with HCA. HCA activates E-cadherin and downregulates highly overexpressed N-cadherin. 

The macrolide compound latrunculin A isolated from the Red Sea sponge *Negombata magnifica* exhibits anti-invasive activity against PC-3 cells [20]. Halichondramide is a trisoxazole-containing macrolide extracted from *Chondrosia corticata* that modulates prostate cancer-related biomarkers such as E-cadherin, N-cadherin, MMP2, and MMP9 at both transcriptional and transitional levels [21].

Epithelial to mesenchymal transition (EMT) biomarkers indicate the metastatic characteristics of prostate cancer [51]. Spongistatin 1 is a macrocyclic lactone derived from the marine sponge *Spongia* sp., that has been shown to induces apoptosis and caspase independent cell death in DU-145 cells [52]. In addition, Spongistatin 1 also upregulates BIM, pro-apoptotic BCL-2 family member BIM, by acting on both the microtubular complex and the antiapoptotic MCL-1. BIM is an important genetic factor that plays a role in upregulating caspase-independent apoptotic signaling pathways executed by mitochondria in prostate cancer [22]. Marine-sponge-derived furanosesterterpene furospinosulin-1 has selective antiproliferative activity against DU-145 cells under hypoxic conditions [23].

Sodwanone and yardenones derived from *Axinella* sp. inhibited PC-3 cells by deactivating HIF-1 [24]. The glycerol ether niphatenone B is a natural product that leads to the development of castration-recurrent prostate cancer that has been isolated from crude methanolic extracts of *Niphates digitalis*. It induces proliferation of LNCaP cells but not PC-3 cells. Consequently, there is no functional AR support against target-specific anti-proliferation. Niphatenone B prominently binds the activation function-1 region of the AR N-terminus domain (NTD) [25]. Finally, agelasine B has been isolated from the marine sponge *Agelas clathrodes.* This compound has been shown to inhibit the viability of PC-3 cells. It significantly reduces the Ca^2+^ concentration in these cells and induces the fragmentation of DNA [26].

### 3.4. Marine Algae

#### 3.4.1. Cyanobacteria

Cyanobacterium (marine blue algae) are a diverse group of prokaryotic organisms. Cryptophycin 52 is a naturally macrocyclic anticancer compound isolated from the marine cyanobacteria *Nostoc* spp. [27].

A new cyclic depsipeptide, lagunamide C was isolated from the marine cyanobacterium, *L. majuscula*, collected from Pulau Hantu Besar, Singapore. Lagunamide C was tested against PC3 cells, with an IC_50_ of 2.6 nM. It also possesses significant antimalarial properties [28].

Cytotoxic peptides like dolastatins isolated from *Dolabella auricularia* and their synthetic analogs dolastatin 10 in symploca and its non-cyanobacterial analog dolastin are responsible for cell cycle arrest in the G2/M phase [29].

Marine cyanobacteria-derived compounds can induce the alteration of caspases and activate the pathway to induced cell death. Caspase-3 is the most well-known caspase in the apoptosis of prostate cancer cell lines. C-phycocyanin (C-PC) s isolated from the *Limnothrix* sp. cyanobacterium has previously been shown to have anticancer properties. We found that only 10% of a typical dose of the topotecan (TPT) anticancer drug combined with C-PC killed cancer cells at a higher rate than that of TPT being used alone at full dose. We also detected an increased magnitude of the increased activities of caspase-9 and caspase-3 when these two compounds were used in combination [30].

The BCL-2 protein family acts as an important regulator of apoptosis in prostate cancer. Cryptophycin 52 promotes BCL-2 and BCL-xL phosphorylation in several prostate cancer cell lines including PC-3, LNCaP, and DU-145 [27].

Iejimalide B, a marine macrolide, was first extracted from the tunicate *Eudistoma cf. rigida*. Iejimalide B is active in both LNCaP and PC-3 cell lines in the nanomolar range, but the effects on the two cell lines differed significantly. One experiment showed that iejimalide B doses below 30 nM induced cell cycle arrest in G0/G1 and cell death at doses at and above 50 nM in LNCaP cells, but neither of these doses induced apoptosis in PC-3 cells after 72 h [31].

#### 3.4.2. Chlorophytes

Chlorophyta is a group of green photosynthetic algae. Most seaweeds are classified as marine chlorophytes and are an important source of vitamins and minerals; they are also promising for their activities against prostate cancer [53]. There has been some research on green algae, which have isolated several potential anticancer compounds. For example, 14-keto-stypodiol diacetate (SDA) extracted from the seaweed *Stypopodium flabelliforme* inhibits cell growth and tumor invasion in DU-145 cells. The studies suggest that this novel derivative from a marine natural product induces the mitotic arrest of tumor cells, an effect that could be associated with alterations in the normal microtubule assembly process. In addition, a salient feature of this compound is that it affects protease secretion and in vitro invasive capacity, both properties of cells from metastases. The secretion of plasminogen activator (u-PA) and the capacity of DU-145 cells to migrate through a Matrigel-coated membrane was significantly inhibited in the presence of micromolar concentrations of SDA. [33]. *Haematococcus pluvialis* is a rich source of carotenoid astaxanthin that is an efficient promoter of antioxidants and apoptosis by inhibiting NF-kappa B, which subsequently inhibits growth in prostate cancer cell lines [34,54].

KF is a significant bioactive compound isolated from *Elysia rufescens*; the actual source of kahalalide is believed to be *Bryopsis* sp. The compound triggered oncogenesis in a prostate cancer cell line. Therefore, KF induced lysosomal and cell membrane permeability and induced apoptosis by inhibiting the PI3K/AKT pathways [55].

#### 3.4.3. Rhodophyta

Rhodophyta, known as red algae, is primarily found in the sea. However, there is not much evidence on the use of red algae extracts as a drug in prostate cancer treatment. Bromophycolides C-I has been isolated from *Callophycus serratus* and has cytotoxic activity against a wide range of cancer cells. Among them, the effect of Bromophycolides D is the most significant. [32].

#### 3.4.4. Phaeophyta

Phaeophyta (brown algae) produce complex diterpenoids and metabolites of mixed terpenoid-aromatic origins. Previous research showed that many of these compounds could be potent antibiotic, antifungal, antiviral, or anticancer agents [56]. They can be isolated from *Cladosiphon novaecaledoniae*, *Undaria pinnatifida,* and other species of brown algae [57]. Fucoidan inhibited PC-3 cells and activated intrinsic and extrinsic apoptosis [35]. This apoptosis was followed by extracellular signal-regulated kinase mitogen-activated protein kinase (ERK1/2 MAPK) and p38 MAPK inactivation. Furthermore, it inactivated phosphatidylinositol 3-kinase (PI3K)/Akt. In addition, p21Cip1/Waf was upregulated following the application of fucoidan. Therefore, fucoidan downregulates E2F-1 cell-cycle-related proteins and upregulates the Wnt/β-catenin signaling pathway. GSK-3β protein activation decreased the β-catenin level and c-MYC and cyclin D1 expression in PC-3 cells [35].

Transforming growth factor β (TGFβ) and receptors (TGFRs) play an important role in the EMT of cancer cells. In one study, fucoidan prominently reversed TGFR-induced EMT morphological changes [36]. Therefore, fucoidan upregulates epithelial markers and downregulates mesenchymal markers as well as decreasing the expression of the transcriptional repressors snail, slug, and twist in prostate cancer cells.

### 3.5. Marine Diatoms

To date, few natural bioactive products have been derived from diatoms despite the abundance of diatoms. Fucoxanthin is an important marine compound in prostate cancer treatment that was isolated from *Sargassum* sp. Fucoxanthin inhibits the growth of LNCaP cells [37]. A growth inhibitory effect was shown by the induction of GADD45A and G1 cell cycle arrest. Fucoxanthin is a highly conjugated natural compound relatively safe for use as an antitumor compound in prostate cancer [38].

Ingested fucoxanthin was reportedly deacetylated in the intestinal lumen and transported via blood in White Leghorn which was fed the brown seaweed *F. serratus*; thus, fucoxanthinol was present as one of the main carotenoids in the egg yolks [58]. Asai et al. investigated the biotransformation of fucoxanthinol in ICR mice, reporting an unknown metabolite which was previously found in the marine tunicate *Amaroucium pliciferum* that was identified as amarouciaxanthin A. Both fucoxanthinol and amarouciaxanthin A reduced the viability of PC-3 cells, with 50% inhibitory concentrations of fucoxanthin, fucoxanthinol, and amarouciaxanthin A of 3.0, 2.0, and 4.6 μM [39], respectively. However, there are few studies on this topic.

### 3.6. Marine Diatom Metabolites

Many novel and physiologically active natural organic compounds have been isolated from soft corals, and further research on these complexes is essential for both the development of marine drugs and the search for new drugs. Five new eunicellin diterpenes, pachycladins A–E (1–5), were isolated from the Red Sea soft coral *Cladiella pachyclados*. Some of the new metabolites exhibited significant anti-invasive activity in PC-3 cells [40]. New metabolite 1 has been isolated from the marine soft coral *Sarcophyton ehrenbergi* along with the known diterpenoids 2 and 3 and cholesterol 4. All of these compounds showed moderate anticancer activity. (*S*)-1 showed modest activity against DU145 cells in the 106–161 mM range. Its synthetic enantiomer (*R*)-1 demonstrated better cytotoxicity against DU145 cells, with an IC_50_ of 77.2 ± 2.5 mM. The naturally obtained membrane 3 compound exhibited good potency against DU145 cells, with an IC_50_ of 75.0 ± 3.8 mM [41].

### 3.7. Holothurians

Holothurians (sea cucumbers) are marine invertebrates that have been used in traditional Asian medicine for centuries [59]. Triterpene glycoside frondoside A (FrA) was initially isolated from an extract of the edible sea cucumber *Cucumaria frondosa.* The FrA compound showed high efficacy and low toxicity in human prostate cancer cells, including cell lines with resistance to standard therapies. Its unique combination of properties includes the simultaneous induction of apoptosis coupled with cell cycle arrest and inhibition of pro-survival autophagy, as well as potential immune modulatory effects [42].

12-MTA inhibited prostate cancer cell lines. PI staining showed that 12-MTA caused PC-3 cell death through the induction of apoptosis, in which caspase-3 may play a role. At relevant biological concentrations, 12-MTA can selectively inhibit the formation of 5-hydroxyeicosatetraenoic acid (5-HETE), a metabolite of 5-lipoxygenase. This agent may be a novel adjunctive therapy for selected malignancies including prostate cancer [43].

## 4. Conclusions

The resistance of marine compounds to prostate cancer has been recognized by academics at home and abroad. The study of the anti-prostate cancer effects of marine compounds is exploring their direct effects on tumor cells by boosting host immune function.

In conclusion, marine compounds have significant potential as anticancer drug compounds. In recent years, the incidence of prostate cancer has been increasing. From an application standpoint, it is difficult to rely on the compounds extracted from land-grown animals and plants to meet clinical needs. Not only is it difficult to collect large numbers of terrestrial animals and plants as it cannot be multiplied, but since some are endangered species, there is a problem of resource competition. Marine sources can be beneficial to prostate cancer research. However, there has been little research on this topic. The organisms studied in the assessment of the anticancer aspects of marine compounds represent only a tiny fraction of the millions of marine creatures. Marine flora exists in large quantities in nature, and many anticancer bioactive compounds have been isolated from them.

This review discussed a number of marine-derived compounds that are related to prostate cancer. Full elucidation of the anti-cancer mechanisms of these compounds, including the clear structure-activity relationship between these compounds and dose-effect may simplify the extraction process. Reasonable pharmacological screening and clinical observation of marine compounds offer promising leads for the development of anticancer drugs or anticancer adjuvants. Marine compounds can play their due role in improving the quality of life of patients with prostate cancer. Further research on these compounds is required for the development of new anti-prostate cancer drugs.

## Figures and Tables

**Table 1:** Marine sources and compounds with potential for anti-prostate cancer drug development.

Source Group	Compounds	Structure	Biological Activity on Prostate Cancer	References
Bacteria	Kahalalide F	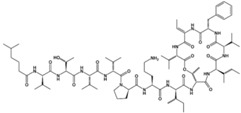	Cytotoxicity (IC_50_: 0.07 μM in PC-3 cells; 0.28 μM in DU-145 cells) 50% of PSA decline for ≥4 weeks at 80 μg/kg/day in clinical trial	[14] [15]
Marine fungi	Demethoxyfumitremorgin C	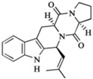	Inhibition of proliferation (50% inhibition at 100 μM in PC-3 cells)	[16]
	Apochalasin V	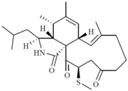	Cytotoxicity (IC_50_: 30.4 μM in PC-3 cells)	[17]
Marine sponges	Rhizochalin	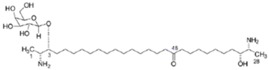	Cytotoxicity (IC_50_: 16.55 μM in PC-3 cells, IC_50_: 10.75 μM in DU-145 cells, IC_50_: 7.88 μM in LNCaP cells, IC_50_: 7.37 μM in 22Rv1 cells, IC_50_: 5.81 μM in VCaP cells)	[18]
	Rhizochalinin	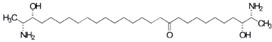	Cytotoxicity (IC_50_: 1.14 μM in PC-3 cells, IC_50_: 1.05 μM in DU-145 cells, IC_50_: 1.69 μM in LNCaP cells, IC_50_: 0.87 μM in 22Rv1 cells, IC_50_: 0.42 μM in VCaP cells)	[19]
	latrunculin A	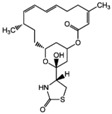	Inhibition of invasion (23% inhibition at 100 nM in PC-3 cells)	[20]
	Halichondramide	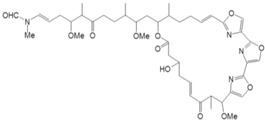	Cytotoxicity (IC_50_: 0.81 μM in PC-3 cells)	[21]
	Spongistatin 1	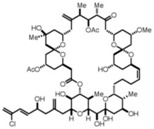	Inhibition of proliferation (50% inhibition at 500 pmol in LNCaP cells)	[22]
	Furospinosulin-1	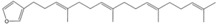	Inhibition of proliferation (60% inhibition at 100 μM in DU-145 cells)	[23]
	Sodwanone and Yardenone	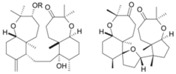	Inhibition of HIF-1α expression at 15 μM in PC-3 cells	[24]
	Niphatenone B	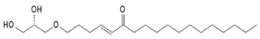	Inhibition of proliferation (90% inhibition at 250 μM in LNCaP cells)	[25]
	Agelasine B	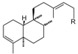	Cytotoxicity (IC_50_: 0.04 μg/mL in DU-145 cells)	[26]
Cyanobacteria	Cryptophycin 52	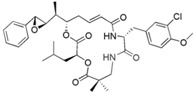	Apoptosis (40% at 250 μg/mL in LNCaP cells)	[27]
	Lagunamide C	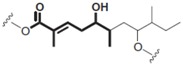	Cytotoxicity (IC_50_: 2.6 nM in PC-3 cells)	[28]
	Dolastatins	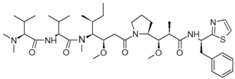	Cell cycle arrest (G2/M arrest in DU-145 cells)	[29]
	C-phycocyanin (C-PC)	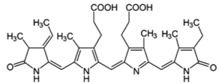	Inhibition of proliferation (30% inhibition at 500 μg/mL in LNCaP cells)	[30]
	Iejimalide B	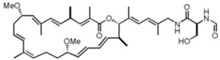	Cell cycle arrest (G0 / G1 arrest in LNCaP cells)	[31]
Rhodophyta	Bromophycolide D	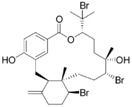	Cytotoxicity (IC_50_: 9.0 μM in PC-3 cells)	[32]
Chlorophyta	14-keto-stypodiol diacetate (SDA)	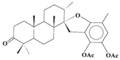	Cytotoxicity (IC_50_: 2.7 μM in DU145 cells)	[33]
	Astaxanthin	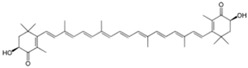	Inhibition of proliferation (38% inhibition at 0.01 μg/mL in LNCaP cells)	[34]
Phaeophyta	Fucoidan	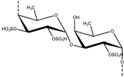	Apoptosis (15.2% at 10 μg/mL, 29.8% at 50 μg/mL, 39.3% at 100 μg/mL, and 45.1% at 200 μg/mL in PC3 cells)	[35,36]
Marine diatoms	Fucoxanthin	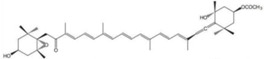	Inhibition of proliferation (50% inhibition at 2.5 μM in LNCaP cells)	[37,38]
	Fucoxanthin, Fucoxanthinol, and Amarouciaxanthin A	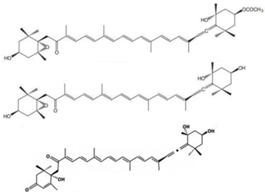	Cytotoxicity (IC_50_: 2.0–4.6 μM in PC-3 cells)	[39]
Corals	Pachycladins A–E	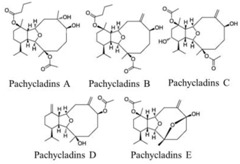	Inhibition of invasion (87% inhibition at 50 μM in PC-3 cells)	[40]
	Metabolite 1 from *Sarcophyton ehrenbergi*, synthetic enantiomer (*R*)-1	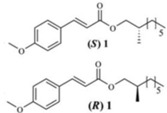	Cytotoxicity ((*S*)-1 (IC_50_: 161 mM in DU-145 cells); (*R*)-1 (IC_50_: 77.2 in DU145 cells)	[41]
Holothurians	Frondoside A	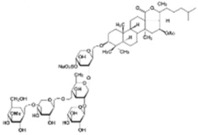	Cell cycle arrest (G2/M-phase at 0.5 µM in PC-3 cells)	[42]
	12-methyltetradecanoic acid	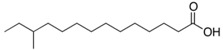	Cytotoxicity (IC_50_: 35.48 μg/mL in DU-145 cells, IC_50_: 20.45 μg/mL in PC-3 cells)	[43]
